# Physical presence of chemical synapses is necessary for turning behavior of anterograde synaptic vesicles at the branch point of PLM neurons in *C. elegans*

**DOI:** 10.17912/micropub.biology.001204

**Published:** 2024-06-25

**Authors:** Amruta Vasudevan, Sandhya P. Koushika

**Affiliations:** 1 Department of Biological Sciences, Tata Institute of Fundamental Research, Mumbai, Maharashtra, India

## Abstract

Neurons exhibit complex branched axonal morphologies in both vertebrate and invertebrate systems, and show heterogeneity in the distribution of synaptic cargo across multiple synapses. It is possible that differences in transport across multiple branches contribute to the heterogeneity in cargo distribution across multiple synapses. However, the regulation of transport at axonal branch points is not well understood. We demonstrate that branch-specific transport of synaptic vesicles is dependent on the existence of a connection between the branch and synapses. The loss of this connection causes an immediate decrease in branch-specific transport of synaptic vesicles in the PLM neuron of *C. elegans* .

**Figure 1. pre-SVs do not turn into the synaptic branch upon loss of connection to chemical synapses in the C. elegans PLM neuron:**
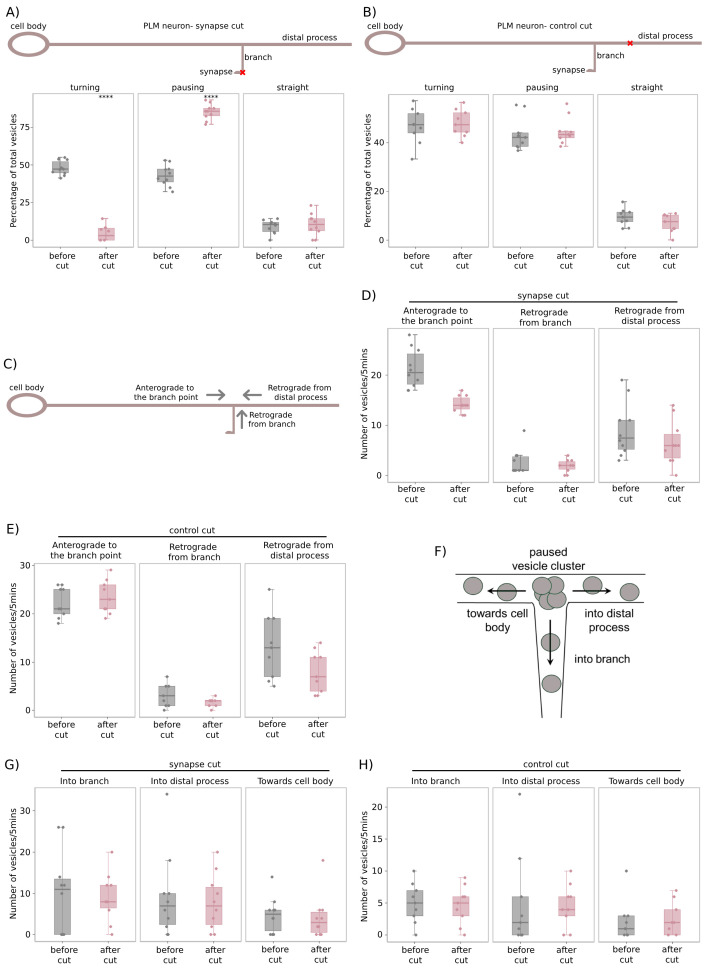
For all plots shown in the figure, individual data points are represented as solid circles. The summary statistics are depicted using a boxplot with whiskers. Upper whisker = minimum(maximum value in the distribution, Q3 + 1.5 * IQR) and lower whisker = maximum(minimum value in the distribution, Q1 – 1.5*IQR), where IQR is inter-quartile range. A) Schematic of the *C. elegans* PLM neuron, representing the laser ablation paradigm for the ‘synapse cut'. The red cross marks the site at which laser ablation is performed, such that the connection between the chemical synapses and the synaptic branch is removed. Proportion of ‘turning', ‘pausing', and ‘straight' vesicles is plotted as a percentage of total anterograde pre-SVs that reach the branch point in wild type- *jsIs821* animals. N(before cut)=10 animals, n(before cut- ‘turning')=103 vesicles, n(before cut-‘pausing')= 90 vesicles, n(before cut-‘straight')= 20 vesicles. N(after cut) =10 animals, n(after cut-‘turning')=6 vesicles, n(after cut-‘pausing')= 121 vesicles, n(after cut-‘straight')= 15 vesicles. Wilcoxon rank sum test with continuity correction conducted to test for statistical significance, ****p<0.0001. All comparisons are made to the ‘before cut' category. B) Schematic of PLM neuron, representing the laser ablation paradigm for the ‘control cut'. The red cross marks the site at which laser ablation is performed along the distal process. This site is chosen such that its distance from the branch point is roughly equal to the length of the PLM synaptic branch (~30µm). Proportion of ‘turning', ‘pausing', and ‘straight' vesicles is plotted as a percentage of total anterograde pre-SVs that reach the branch point in wild type- *jsIs821* animals. N(before cut)=9 animals, n(before cut- ‘turning')=95 vesicles, n(before cut-‘pausing')=87 vesicles, n(before cut-‘straight')=19 vesicles. N(after cut) =9 animals, n(after cut-‘turning')=101 vesicles, n(after cut-‘pausing')=95 vesicles, n(after cut-‘straight')=15 vesicles. Wilcoxon rank sum test with continuity correction conducted to test for statistical significance. All comparisons are made to the ‘before cut' category and are not statistically significant. C) Schematic of the PLM neuron depicting the different categories of pre-SVs transported at the branch point, namely, i) anterograde pre-SVs transported from the cell body to the branch point, ii) retrograde pre-SVs transported from the synaptic branch to the branch point, and iii) retrograde pre-SVs transported from the distal process to the branch point. D) Total flux of pre-SVs reaching the branch point in the anterograde and retrograde directions over a period of 5mins in the ‘synapse cut' paradigm. N(before cut)=10 animals, n(before cut- ‘anterograde')=213 vesicles, n(before cut- ‘retrograde from branch')=26 vesicles, n(after cut- ‘anterograde')=142 vesicles, n(after cut-‘retrograde from branch')=19 vesicles. Wilcoxon rank sum test with continuity correction conducted to test for statistical significance, all comparisons are non-significant. All comparisons are made to the ‘before cut' category. E) Total flux of pre-SVs reaching the branch point in the anterograde and retrograde directions over a period of 5mins in the ‘control cut' paradigm. N(before cut)=9 animals, n(before cut- ‘anterograde')=201 vesicles, n(before cut- ‘retrograde from distal process')=119 vesicles, n(after cut- ‘anterograde')=211 vesicles, n(after cut-‘retrograde from branch')=72 vesicles. Wilcoxon rank sum test with continuity correction conducted to test for statistical significance, all comparisons are non-significant. All comparisons are made to the ‘before cut' category. F) Schematic of the PLM branch point depicting the mobilization of pre-SVs from the paused vesicle cluster at the branch point into different compartments of the neuron. G) Total flux of pre-SVs mobilizing from the paused vesicle cluster at the branch point over a period of 5mins in the ‘synapse cut' paradigm. N(before cut)=10 animals, n(before cut-‘Into branch')=50 vesicles, n(before cut-‘Into distal process')=46 vesicles, n(before cut-‘Towards cell body')=24 vesicles. N(after cut) =10 animals, n(after cut-‘ Into branch')=45 vesicles, n(after cut-‘Into distal process')=39 vesicles, n(after cut-‘Towards cell body')=21 vesicles. Wilcoxon rank sum test with continuity correction conducted to test for statistical significance, all comparisons are non-significant. All comparisons are made to the ‘before cut' category. H) Total flux of pre-SVs mobilising from the paused vesicle cluster at the branch point over a period of 5mins in the ‘control cut' paradigm. N(before cut)=9 animals, n(before cut-‘Into branch')=45 vesicles, n(before cut-‘Into distal process')=46 vesicles, n(before cut-‘Towards cell body')=20 vesicles. N(after cut) =9 animals, n(after cut-‘Into branch')=42 vesicles, n(after cut-‘Into distal process')=41 vesicles, n(after cut-‘Towards cell body')=22 vesicles. Wilcoxon rank sum test with continuity correction conducted to test for statistical significance, all comparisons are non-significant. All comparisons are made to the ‘before cut' category.

## Description

Chemical synapses are known to play a role in remodeling of axonal branches (Alsina et al., 2001, Javaherian & Cline, 2005, Panzer et al., 2006, Ruthazer et al., 2006) . Presynaptic sites that carry a greater number of clustered synaptic vesicles, or display synaptic activity, are shown to stabilize the associated axonal branches (Ruthazer et al., 2006) . Since chemical synapses regulate the formation and structural integrity of axonal branches, they may also affect the transport of cargo along these branches. The regulation of vesicular transport and distribution by *en passant* chemical synapses is a field of active study, and several studies suggest that presynaptic boutons capture moving synaptic vesicles through various AZ proteins and signaling kinases, which likely oppose the mobility of synaptic vesicles locally (Wu et al., 2013, Edwards et al., 2015, Edwards et al., 2018, Lipton, Maeder, and Shen, 2018, Morrison et al., 2018) . In contrast to *en passant* synapses, little is known about the regulation of branch-specific transport of synaptic cargo by terminal synapses, which are located at the end of axonal branches. Several studies show that increased synaptic/growth cone activity in axonal branches is strongly correlated with increased synaptic cargo transport along those branches (Aletta & Goldberg, 1984, Schacher, 1985, Goldberg & Ambron, 1986, Goldberg & Schacher, 1987, Schacher et al., 1999, Ruthel & Hollenbeck, 2003, Lyles, Zhao, & Martin, 2006, Tymanskyj, Curran, and Ma, 2022) . However, the mechanism of this synapse-mediated regulation of branch-specific transport *in vivo* is not understood at all. It is unclear whether synapses regulate branch-specific cargo transport simply by stabilization of the branch or through additional mechanisms.

It has been proposed that terminal synapses send feedback signals to the neuronal cell body in order to maintain transport of synaptic cargo in the associated branch, and that the strength of this signal likely scales with the synaptic strength of the associated branch (Aletta & Goldberg, 1982, Aletta & Goldberg, 1984) . A recent study demonstrated that, following synaptogenesis, an unidentified retrograde signal from the postsynaptic neuron can trigger large-scale transcriptional and translational changes in the presynaptic neuron, which in turn increases the flux of bidirectional mitochondrial transport in the presynaptic neuron (Badal et al., 2019) . However, the source and nature of these retrograde signals is unknown. Several recent studies examine the ability of synapses to capture transiting vesicles locally (Wong et.al.,2012, Bulgari et.al.,2014). Known instances of retrograde signaling occur over shorter distances at individual synaptic boutons (Zhao & Nonet, 2000, Doi & Iwasaki, 2002, Chang et al., 2011, Hsieh et al., 2014) , and it is rare to find studies which discuss the existence of long-range synaptic signals that act from a distance to direct vesicles towards themselves. We have previously shown that branch-specific transport of synaptic vesicles is regulated by the levels of the anterograde motor UNC-104 /Kinesin-3 (Vasudevan et al., 2024) in the PLM neuron of *C. elegans* . It is possible that long-range retrograde signaling from synapses, if it exists, regulates branch-specific transport by acting on the motor-cargo complex and/or the cytoskeleton. Chemical synapses in the *C. elegans* PLM neuron play a role in stabilizing the synaptic branch, similar to studies conducted in vertebrate neurons as discussed earlier. It has been observed that removing the connection between the synaptic branch and the chemical synapse causes the synaptic branch to retract into the main process over a timescale of 24hrs (Wu et al., 2007) . Since the PLM synapse is necessary for maintenance of the synaptic branch, we investigated whether it regulates the branch-specific transport of synaptic vesicle precursors (pre-SVs).

We have previously shown that anterograde pre-SVs either turn into the synaptic branch, pause at the branch point, or go straight into the distal process of the PLM neuron (Vasudevan et al., 2024) . In this study, we used a laser ablation paradigm as described in (Figs. 1A & 1B) to investigate the role of the PLM chemical synapses in regulating branch-specific transport of pre-SVs. To visualize transport of pre-SVs, we used transgenic animals expressing GFP:: RAB-3 specifically in the touch receptor neurons (TRNs). Although this transgene overexpresses RAB-3 in TRNs, we did not observe any defects in synaptic vesicle transport due to the overexpression. The PLM synaptic branch was ablated distally, leaving most of the branch process intact while severing its connection to the cluster of chemical synapses ( Fig. 1A ). This was termed as a ‘synapse cut'. At the PLM branch point, transport of GFP::RAB-3-labelled pre-SVs was examined for 5 minutes prior to the ablation, and for 5 minutes immediately after the ‘synapse cut'. Prior to the ablation, ~40-50% of anterogradely moving pre-SVs turned into the synaptic branch, ~40% paused at the branch point, and the remaining ~10% went straight into the distal process ( Fig. 1A, Extended Figure 1B), consistent with our prior observations (Vasudevan et al., 2024) . Immediately following the ‘synapse cut', the proportion of anterograde pre-SVs turning into the synaptic branch reduced significantly to ~5-10%, a majority (~80%) paused at the branch point, while the proportion going straight into the distal process was unchanged (~10%) ( Fig. 1A, Extended Figure 1B). To ensure that the changes observed in anterograde pre-SV transport at the PLM branch point were not artefacts of laser ablation, a ‘control cut' was performed ( Fig. 1B ), where the PLM distal process was ablated at approximately the same distance from the branch point as the average length of the PLM synaptic branch (~30µm). The proportion of anterograde pre-SVs that turn into the synaptic branch, pause at the branch point, and go straight into the distal process were not significantly different from the uncut controls ( Fig. 1B, Extended Figure 1C).

In both the ‘synapse cut' and ‘control cut', we tested whether vesicular transport was altered in the ablated process by examining the number of pre-SVs transported in both the anterograde and retrograde directions along the synaptic branch and distal process (Figs. 1C & 1F). Anterograde transport in the synaptic branch comprises pre-SVs that i) turn into the branch, or ii) mobilize from the paused vesicle cluster at the branch point into the branch ( Fig. 1F ), while pre-SVs moving from the branch back to the branch point contribute to retrograde transport in the branch ( Fig. 1C ). Similarly, anterograde pre-SVs i) going straight across the branch point or ii) mobilizing from the paused vesicle cluster into the distal process contribute to anterograde transport in the distal process ( Fig. 1F ), while retrograde transport comprises pre-SVs moving from the distal process back to the branch point ( Fig. 1C ). We found that the flux of retrograde pre-SVs originating from the synaptic branch and the distal process did not differ significantly in both ablation paradigms (Figs. 1D & 1E, Movies 1,2,3,&4). Similarly, the proportion of pre-SVs mobilizing from the paused vesicle cluster to the synaptic branch and distal process did not significantly change in both ablation paradigms (Figs. 1G & 1H, Movies 1,2,3,&4). It is important to examine how the microtubule distribution is perturbed immediately after the ablation paradigms, however our observation that vesicular transport in the ablated process is not significantly altered suggests that the microtubules are not perturbed at these timescales. In summary, the loss of PLM synapses has an immediate and specific effect on the turning behavior of anterograde pre-SVs, suggesting that chemical synapses likely send out continuous signals that regulate vesicle entry into the synaptic branch. These signals are likely sensed by the axonal transport machinery of pre-SVs and responded to almost immediately (~1min), as anterogradely moving pre-SVs lose their preference for turning into the synaptic branch minutes after the connection of the synaptic branch to the chemical synapses is severed.

## Methods

Maintenance of *C. elegans* strains

*C. elegans* strains were grown at 20°C on Agar plates made with Nematode Growth Medium and seeded with *E. coli* OP50 (Brenner, 1974). Wormbase ( http://www.wormbase.org ) was used as a reference for information on phenotypes of different strains (Sternberg et al., 2024) . Animals belonging to the young adult stage were picked for imaging and laser ablation from uncrowded, uncontaminated plates.

Time-lapse image acquisition

Live animals were anesthetized in 3mM Tetramisole in M9 buffer on 5% agarose pads. Imaging was conducted on an Olympus IX83 inverted fluorescence microscope, integrated with the Yokogawa CSU-X1 spinning disc scan head from Perkin Elmer UltraVIEW, and the Hamamatsu ImagEM C9100-13/14 EMCCD Camera, using a 488nm diode-pumped solid state laser. Software used for image acquisition was Volocity 6.0 by Perkin Elmer. Images of the PLM branch point were acquired in the Green channel at a frame rate of 5 frames per second (fps), using a UPLSAPO 100XO, 1.4 N.A. oil immersion objective. Typically, the length of the PLM process imaged spans ~70-80μm, and the length of the PLM synaptic branch imaged spans ~5-20μm depending on the orientation of the immobilized animal. Time-lapse movies were taken for a duration of 10mins.

Laser-based axotomy of the PLM synaptic branch and distal process

Laser ablation experiments were performed using a 355nm Ultraviolet range pulsed nanosecond laser (Minilite Series, Flashlamp pumped, Q-Switched, Nd:YAG) operated in low energy mode at a repetition rate of 10, with a manual trigger. Time-lapse fluorescence images of the PLM branch were acquired at 5fps, using a 100X/1.4 NA oil objective on the Olympus IX83 microscope, integrated with the Yokogawa CSU-X1-A3 spinning disc and Hamamatsu ImagEM C9100-13/14 EMCCD Camera (by Perkin Elmer), using a 488nm diode-pumped solid state laser. 5min movies were acquired prior to, and immediately after the ablation of the PLM synaptic branch/distal process.

Kymograph generation and annotation of pre-SV trajectories

All image panels used for representation and analysis of time lapse movies were generated using FIJI (ImageJ v1.52p). Experimental kymographs were generated using the ‘MultipleKymograph' plugin. Plugins were downloaded from the NIH website using the links http://www.rsbweb.nih.gov/ij/ and http://www.emblheidelberg.de/eamnet/html/bodykymograph.html.

In the kymograph, pre-SVs moving in the retrograde direction (towards the cell body), and anterograde direction (away from the cell body) appear as sloped lines, while stationary cargo appear as vertical lines. A cargo is counted as moving if it has been displaced by at least 3 pixels in successive time frames.

For each movie of the PLM branch point, a ‘branch kymograph' and ‘straight kymograph' are generated as follows:

a) Branch kymograph is generated by tracing a curved segmented line along the neuron from the branch to the pre-branch main process

b) Straight kymograph is generated by tracing a straight segmented line from the distal process to the pre-branch region along the main process

Since the branch and straight kymographs both share the same pre-branch region and only differ from each other beyond the branch point, a comparison of vesicle trajectories in both kymographs helps us identify the branch point. Consequently, a vesicle trajectory that starts in the pre-branch region and crosses the branch point in the branch kymograph is categorized as a ‘turning vesicle'. A vesicle trajectory that starts in the pre-branch region and crosses the branch point in the straight kymograph is categorized as a ‘straight vesicle'. A vesicle trajectory that starts in the pre-branch region and stops at the branch point in both the branch and straight kymographs is categorized as a ‘pausing vesicle'.

Quantification and statistical analysis

Kymographs manually annotated with pre-SV trajectories are used to identify and quantify turning, pausing, straight vesicles, and pre-SVs mobilizing from the branch point using custom ImageJ macros ‘SegmentsBranch.ijm', ‘SegmentsStraight.ijm', ‘AnalysisBranch.ijm', ‘AnalysisStraight.ijm' developed for this analysis. These codes are available at the GitHub repository link https://github.com/amruta2612/Fiji-macros . Wilcoxon rank sum test (non-parametric) with continuity correction was used on data sets that did not pass the test of normality and the distributions across different categories were varied. p-value<0.0001 is marked as ****. All statistical tests were conducted in R.

## Reagents

**Table:** 

S. No.	Strain number	Genotype	Reference
1	NM2689	*jsIs821* [ *mec-7* p:: *gfp* :: *rab-3* ]	Bounoutas et al., 2009

## Extended Data

Description: Raw data for figure 1 . Resource Type: Dataset. DOI: 10.22002/b2jqz-qdw65

Description: pre-SV transport at the branch point of the PLM neuron in jsIs821 young adult animals before ‘synapse cut’. Scale bar=5µm.. Resource Type: Audiovisual. DOI: 10.22002/bh4wj-t6787

Description: pre-SV transport at the branch point of the PLM neuron in jsIs821 young adult animals immediately after ‘synapse cut’. Scale bar=5µm.. Resource Type: Audiovisual. DOI: 10.22002/rz0np-0gy12

Description: pre-SV transport at the branch point of the PLM neuron in jsIs821 young adult animals before ‘control cut’. Scale bar=5µm.. Resource Type: Audiovisual. DOI: 10.22002/a6m6t-3fr28

Description: pre-SV transport at the branch point of the PLM neuron in jsIs821 young adult animals after ‘control cut’. Scale bar=5µm.. Resource Type: Audiovisual. DOI: 10.22002/942sz-7ta97

Description: Supplementary figure 1 showing kymographs of pre -SV transport before and after both ablation paradigms. Resource Type: Image. DOI: 10.22002/kjd96-1ze77

Description: Legend and caption for Extended Figure1. Resource Type: Text. DOI: 10.22002/x9mw9-bds71
